# Diabetes and Weight in Comparative Studies of Bariatric Surgery vs Conventional Medical Therapy: A Systematic Review and Meta-Analysis

**DOI:** 10.1007/s11695-013-1160-3

**Published:** 2013-12-28

**Authors:** G. Ribaric, J. N. Buchwald, T. W. McGlennon

**Affiliations:** 1 European Surgical Institute, Ethicon Endo-Surgery (Europe) GmbH, Hamburg, Germany; 2 Division of Scientific Research Writing, Medwrite Medical Communications, Maiden Rock, WI USA; 3 Statistical Analysis & Quality of Life Assessment, McGlennon MotiMetrics, Maiden Rock, WI USA; 4 Ethicon Endo-Surgery (Europe) GmbH, MD&D EMEA (Europe, Middle East, Africa), Johnson & Johnson, Hummelsbütteler Steindamm 71, 22851 Norderstedt, Germany

**Keywords:** Bariatric, Metabolic, Comparative, Type 2 diabetes mellitus, T2DM, Systematic review, Meta-analysis, Randomized controlled trial

## Abstract

We performed a meta-analysis of weight loss and remission of type 2 diabetes mellitus (T2DM) evaluated in randomized controlled trials (RCTs) and observational studies of bariatric surgery vs conventional medical therapy. English articles published through June 10, 2013 that compared bariatric surgery with conventional therapy and included T2DM endpoints with ≥12-month follow-up were systematically reviewed. Body mass index (BMI, in kilogram per square meter), glycated hemoglobin (HbA_1C_, in degree), and fasting plasma glucose (FPG, in milligram per deciliter) were analyzed by calculating weighted mean differences (WMDs) and pooled standardized mean differences and associated 95 % confidence intervals (95 % CI). Aggregated T2DM remission event data were analyzed by calculating the pooled odds ratio (POR) and 95 % CI. Random effects assumptions were applied throughout; *I* ^2^ ≥ 75.0 % was considered indicative of significant heterogeneity. Systematic review identified 512 articles: 47 duplicates were removed, 446 failed inclusion criteria (i.e., *n* < 10 per arm, animal studies, reviews, case reports, abstracts, and kin studies). Of 19 eligible articles, two not focused on diagnosed T2DM and one with insufficient T2DM data were excluded. In the final 16 included papers, 3,076 patients (mean BMI, 40.9; age, 47.0; 72.0 % female) underwent bariatric surgery; 3,055 (39.4; 48.6, 69.0 %) received conventional or no weight-loss therapy. In bariatric surgery vs conventional therapy groups, the mean 17.3 ± 5.7 month BMI WMD was 8.3 (7.0, 9.6; *p* < 0.001; *I* ^2^ = 91.8), HbA_1C_ was 1.1 (0.6, 1.6; *p* < 0.001; *I* ^2^ = 91.9), and FPG, 24.9 (15.9, 33.9; *p* < 0.001; *I* ^2^ = 84.8), with significant differences favoring surgery. The overall T2DM remission rate for surgery vs conventional group was 63.5 vs 15.6 % (*p* < 0.001). The Peto summary POR was 9.8 (6.1, 15.9); inverse variance summary POR was 15.8 (7.9, 31.4). Of the included studies, 94.0 % demonstrated a significant statistical advantage favoring surgery. In a meta-analysis of 16 studies (5 RCTs) with 6,131 patients and mean 17.3-month follow-up, bariatric surgery was significantly more effective than conventional medical therapy in achieving weight loss, HbA_1C_ and FPG reduction, and diabetes remission. The odds of bariatric surgery patients reaching T2DM remission ranged from 9.8 to 15.8 times the odds of patients treated with conventional therapy.

**Electronic supplementary material:**

The online version of this article (doi:10.1007/s11695-013-1160-3) contains supplementary material, which is available to authorized users.

## Introduction

“Diabesity,” a term coined by Dr. Ethan Sims in 1973 to denote comorbid obesity and type 2 diabetes mellitus (T2DM) [1], has steadily grown into a global epidemic. The World Health Organization (WHO) estimates the number of overweight adults at >1.6 billion and >400 million who are obese. By 2015, >2.3 billion adults are projected to be overweight with >700 million obese [2]. Globally, >312 million people suffer from T2DM [3], a disease associated with a markedly increased risk of heart disease and stroke, micro- and macrovascular consequences, retinopathy, and kidney failure [4]. While weight loss and its maintenance, by any means, aids in improving and managing T2DM [5, 6], long-term antidiabetic diet compliance is poor even when supported by pharmacotherapy; 50.0 to 90.0 % of patients remain unable to achieve adequate diabetes control [7–9].

In the long-running, prospective, controlled Swedish Obese Subjects (SOS) study, weight loss by conventional medical therapy was associated with T2DM remission of approximately 21.0 % (*n* = 248) at 2 years (compared with 72.0 % remission in postbariatric surgery patients [*n* = 342]) and 12.0 % (*n* = 84) at 10 years (vs 37.0 % bariatric surgery group remission [*n* = 118]) [10], an approximately threefold difference in effective control of diabetes favoring bariatric surgery. Typically, when even excellent weight loss has been achieved by very low calorie diets (VLCDs) and intensive lifestyle programs, neither weight loss nor diabetes resolution has been maintained beyond 1–5 years [10–13] nor has diabetes resolved as rapidly as following most bariatric procedures (i.e., within days to a few weeks [14]). Several series suggest that although the weight-loss effect of bariatric surgery is attenuated in lower body mass index (BMI, in kilogram per square meter) patients [15, 16], surgery may achieve a higher rate of diabetes resolution than conventional medical therapy in patients who are only overweight (BMI ≥ 25–29.9) through those with class III obesity (BMI ≥ 40.0) [17–19]. Diabetes and obesity are progressive, multifactorial diseases; it is probable that bariatric surgery and/or one of the emerging, less-invasive, endolumenal procedures in combination with life-long lifestyle modification may represent an optimum management strategy [11, 20, 21].

Procedures that most effectively reduce weight, such as Roux-en-Y gastric bypass (RYGB) and biliopancreatic diversion, realize 80.0 and 95.0 % hyperglycemia remission, respectively [22], a markedly greater treatment effect than that seen in the majority of studies of conventional dietary and pharmacologic therapy; most patients fail to achieve the goal for glycemic control of <7.0 % glycated hemoglobin (HbA_1C_) prescribed by the American Diabetes Association (ADA) [23, 24]. The etiology of diabetes remission following bariatric surgery is not fully understood. Remission may be engaged by divergent and/or additional mechanisms through bariatric surgery [25], as individual procedures reorganize the gastrointestinal (GI) tract differently, activating varied neurohormonal mechanisms [26, 27]. Preclinical [28] and clinical evidence [19, 29, 30] suggest that improved glycemic control is not linked exclusively with baseline weight or operative weight loss and results from complementary processes [15, 22, 31–33].

Our aim was to assess diabetes and weight outcomes in comparative studies of bariatric surgery vs conventional medical therapy. In preliminary research, we found few directly comparative, level 1, randomized controlled trial (RCT) outcomes (as defined by the Oxford Centre for Evidence-Based Medicine [34]). As a result, we broadened our inclusion criteria to incorporate directly comparative observational studies (OSs). Thus, the current review systematically identified and screened comparative studies of bariatric surgery vs conventional medical therapy in adults with a mean BMI ≥ 25 and subjected the aggregated weight and diabetes data (BMI, HbA_1C_, fasting plasma glucose (FPG), and diabetes remission) to meta-analysis.

## Methods

### Inclusion Criteria and Search Strategy

An electronic literature search and cross-referencing of articles was performed within the following databases: National Library of Medicine PubMed®/MEDLINE®, SpringerLink®, and SciVerse®. The search strategy followed the identification and screening guidelines established by the Preferred Reporting Items for Systematic Reviews and Meta-Analyses (PRISMA) statement [35]. Articles were identified by Boolean combination of keywords: “bariatric surgery,” “metabolic surgery,” “diabetes surgery,” “gastric band,” “sleeve gastrectomy,” “gastric bypass,” “duodenal switch,” “biliopancreatic diversion,” *with* “medical treatment,” “medical therapy,” “conventional treatment,” “conventional therapy,” and “diet.” An additional search using keyword phrases, “bariatric surgery, diabetes mellitus,” and “bariatric surgery, glucose OR insulin OR HbA_1C_ or HOMA,” was run. Limits set to govern the searches stipulated journal articles that featured comparative studies on adult human subjects written in the English language with no beginning date through June 10, 2013.

Compiled article citations were screened by title to exclude duplicates arising from unintentional collection of both e-publications and their follow-on print versions. The unique citations were evaluated by review of abstracts. Articles with *n* < 10 in any study arm, reviews, animal studies, case reports, abstracts, book chapters, kin studies (i.e., reports with overlapping data, or outcomes reported for the same timeframe and/or by the same author group), and Comments or Letters to the Editor were excluded from eligibility. The remaining articles were read in full and assessed by two researchers to ensure that all or a subset of the overweight, mildly obese, or morbidly obese patients in each treatment arm had been diagnosed with T2DM (of any duration) prior to undergoing bariatric surgery or conventional therapy. Finally, articles evaluating fewer than one of the aforementioned T2DM endpoints were excluded from quantitative analysis.

### Defining Diabetes Remission and Data Extraction

The recommended glycemic goal for HbA_1C_ stipulated by the 2009 ADA Standards of Medical Care in Diabetes for adults is <7.0 % with a suggested normal range of 4.0–6.0 % [24]. A variety of definitions of T2DM remission have been used in bariatric surgery and conventional medical therapy studies. In the current analysis, the percentage of patients that achieved T2DM remission, as assessed independently in each included study, characterized T2DM remission rate.

Variable data of interest were extracted from included studies and entered into a dedicated database. Data collection objectives centered on study characteristics (including bariatric procedures, conventional treatments, study designs, and analysis time points); demographic and anthropometric measures (age, gender, BMI [weight (in kilogram), divided by height (in square meter)] [36]); markers of glycemic control (HbA_1C_ and FPG); and T2DM remission rate. Assessment of study quality indicated that a range of diverse medical therapies characterized the non-surgical control groups. In addition, certain studies combined outcome data from multiple bariatric surgery procedures and presented results in the form of a general surgery group vs conventional therapy; no stratification was used. The present authors opted to extend the concept of “grouping” to each study that met inclusion criteria; i.e., if studies reported on more than two arms (e.g., multiple surgery procedures and/or multiple forms of conventional therapy), data were pooled using weighted means and standard deviations to represent summary data for one “combined surgery group” vs one “combined conventional therapy group” per study.

### Statistical Analysis

Data manipulation and analysis were conducted using SPSS® software, version 20.0 (IBM SPSS, Chicago, IL, USA) in conjunction with Comprehensive Meta Analysis 2.2 (Biostat, Englewood, NJ, USA). Body mass index, HbA_1C_, and FPG were analyzed by calculating weighted mean differences (WMDs) and pooled standardized mean differences (SMDs) and associated 95 % confidence intervals (CI). Aggregated T2DM remission event data were analyzed by calculating the pooled odds ratio (POR) and 95 % CI. Random effects assumptions were applied throughout; *I* ^2^ ≥ 75.0 % was considered indicative of significant heterogeneity. [See Electronic Supplementary Material, Appendix 1 including references 37–40 for detailed statistical methodology.]

## Results

### Study Characteristics

**Fig. 1:**
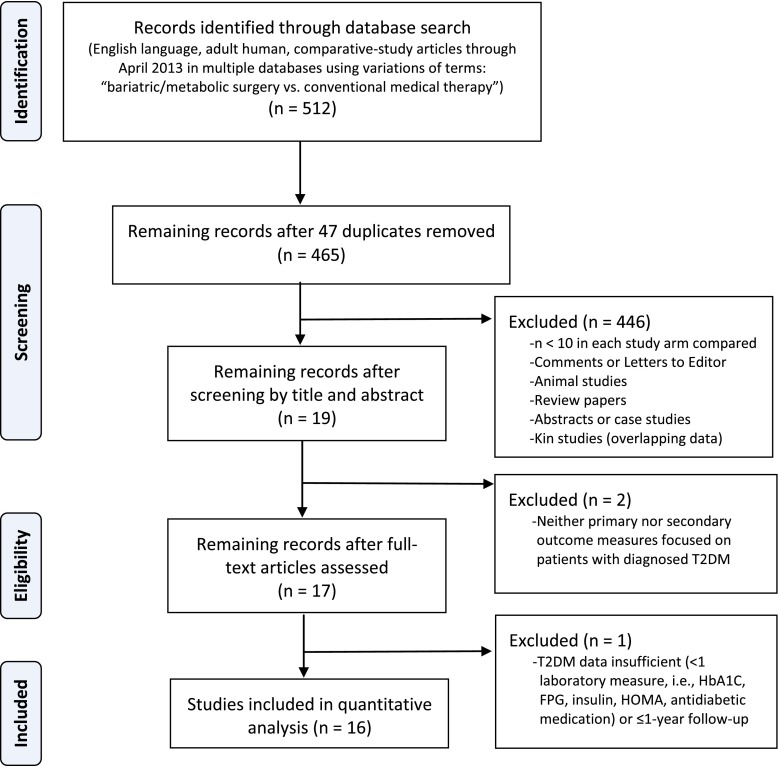
Outcomes of the systematic review of the literature by record identification, screening, and analysis in the Preferred Reporting Items for Systematic Reviews and Meta-Analyses (PRISMA) statement flow diagram

The results of the systematic review are presented in Fig. 1. A total of 512 articles were identified by extensive electronic database search. Forty-seven duplicates were removed. After screening 465 unique citations by title and abstract, 446 failed to meet inclusion criteria and were excluded. The remaining 17 articles were read and assessed for eligibility. Two that provided neither primary nor secondary outcomes related to patients with diagnosed T2DM and one that provided insufficient T2DM data to assess at the 12-month analysis time point were excluded, leaving a final set of 16 articles [41–56] for quantitative analysis.

Characteristics of included studies are described in Table 1. Comparative articles were published over a slightly less than 10-year span, between December 23, 2004 and June 10, 2013, the majority (13/16, 81.3 %) in the last 3.5 years. The respective country of origin (based on first author’s affiliation) and article distribution was: USA, six (38.0 %); Italy, four (25.0 %); Australia, two (12.5 %); Norway, two (12.5 %); Sweden, one (6.0 %); and Korea, one (6.0 %). Study designs included 5 (31.0 %) nonblinded RCTs, 11 OSs, 3 (19.0 %) nonrandomized controlled trials (nRCTs), 5 (31.0 %) prospective comparison studies, and 3 (19.0 %) retrospective database reviews. All studies obtained local institutional or ethics review board protocol approval, 12 mentioned obtaining informed consent, and 8 were nationally registered as clinical trials. Often, more than one WHO weight class was studied in a report: 11 (69.0 %) included morbidly obese patients, 4 (25.0 %) obese, 5 (31.3 %) mildly obese, and 1 (6.3 %) overweight. Our analysis focused on results collected between 12 and 24 months following study commencement. Mean follow-up was 17.3 ± 5.7 months (median 15.0 months). T2DM remission was defined variably across studies; however, the target criteria was always identical for the two arms within each individual study.

**Table 1:** Characteristics of included comparative studies

Study	Country^a^	Bariatric procedure(s)	Conventional weight-loss therapy	Study design + weight class, T2DM variables, IRB, IC, Reg.	Analysis time points (months)
Sjöström et al. [40]	Sweden	LAGB (19.0 %; adjustable and nonadjustable) VBG (68.0 %) RYGB (13.0 %)	Nonstandardized nonsurgical treatment ranging from lifestyle intervention and behavior modification to no treatment	nRCT (“SOS study”) w/contemporaneous subject matching	24; 120
				Morbidly obese patients	
				T2DM variables = FPG, med. use	
				IRB, IC	
				Reg. Swedish Obese Subjects (SOS) registry	
O’Brien et al. [41]	Australia	LAGB in addition to lifestyle modification instruction (i.e., increased exercise + good eating practices)	Intensive weight-loss program including initial VLCD (500 to 550 kcal/day, followed by transition phase), pharmacotherapy, and lifestyle change tailored to patients individually	RCT, nonblinded	24
				Mildly obese patients	
				T2DM variables = FPG, insulin	
				IRB, IC	
				Reg. ACTRN012605000113651	
Dixon et al. [42]	Australia	LAGB in addition to conventional T2DM therapy	Conventional dietary and T2DM therapy administered by diabetologist with focus on lifestyle change	RCT, nonblinded	24
				Mild to morbidly obese patients	
				T2DM variables = FPG, HbA_1C_, HOMA, med. use	
				IRB, IC	
				Reg. ACTRN012605000159651	
Hofsø et al. [43]	Norway	RYGB after following low-calorie diet for 3–6 weeks	Intensive lifestyle intervention through four 1–4 week stays at rehabilitation center specializing in care of morbidly obese patients; cognitive approach to motivate increased activity and normalized eating habits	nRCT (“MOBIL trial”)	12
				Morbidly obese patients	
				T2DM variables = FPG, HbA_1C_, insulin, med. use	
				IRB, IC	
				Reg. NCT00273104	
Adams et al. [44]	USA	RYGB	No structured, monitored weight-loss intervention in either of 2 nonsurgical control groups (1 = nonintervened surgery-seekers; 2 = population-based subjects not seeking surgery)	nRCT	24
				Morbidly obese patients	
				T2DM variables = FPG, HbA_1C_, HOMA-IR, insulin, med. use	
				IRB, IC	
				Reg. NIDDK DK-55006 & NCRR M01-RR00064	
Serrot et al. [45]	USA	RYGB	Routine medical management with nutrition, weight management, and exercise counseling	Retrospective study of surgery recipients and database of matched nonsurgical controls	12
				Mildly obese patients	
				T2DM variables = HbA_1C_, med. use	
				IRB, no Reg.	
Martins et al. [46]	Norway	RYGB	Option of residential intermittent program; commercial weight-loss camp; or hospital outpatient program	Prospective comparison of patients on surgery wait list given option of surgery or 1 of 3 conservative weight-loss programs	12
				Morbidly obese patients	
				T2DM variables = FPG	
				IRB, IC, no reg.	
Iaconelli et al. [47]	Italy	BPD	Individualized medical therapy with conventional weight, exercise, and dietary support; diabetologist available for consult every 3 months	Prospective, matched, open case-controlled trial	12–120
				Morbidly obese patients	
				T2DM variables = FPG, HbA_1C_, T2DM remission, HOMA, insulin	
				IRB, IC, no Reg. study	
				Obese and morbidly obese patients	
				T2DM variables = FPG, HbA_1C_, T2DM remission, med. use	
				IRB, no Reg.	
Scopinaro et al. [48]	Italy	BPD	Routine medical therapy	Prospective study of surgical patients matched with nonsurgical database controls	12
				Overweight and mildly obese patients	
				T2DM variables = FPG, HbA_1C_, HOMA-IR	
				IRB, IC, no reg.	
Leonetti et al. [49]	Italy	LSG	Standard medical therapy with individual lifestyle modification programs, access to diabetologists, dietician, nurse.	Prospective study of surgical patients matched with nonsurgical controls	3; 6; 12; 18
				Obese and morbidly obese patients	
				T2DM variables = FPG, HbA_1C_, med. use	
				IRB, IC, no Reg.	
Mingrone et al. [50]	Italy	RYGB (50.0 %) BPD (50.0 %)	Diet, exercise, lifestyle modification program, including medication optimization and treatment by diabetologist, dietician, nurse—1, 3, 6, 9, 12, and 24 months.	• RCT, nonblinded	24
				• Morbidly obese patients	
				• T2DM variables = FPG, HbA_1C_, med. use, T2DM remission	
				IRB, IC	
				Reg. NCT00888836	
Heo et al. [51]	Korea	RYGB (28.0 %)	Conventional medical therapy, including lifestyle modification, medication optimization, counseling by dietician and exercise practitioner	Retrospective multicenter database	18
		LAGB (27.6 %) LSG (44.4 %)			
Dorman et al. [52]	USA	RYGB (60,1 %) LAGB (20.9 %) DS (18.8 %)	Conventional management by endocrinologist for medication use; lifestyle modification to promote weight loss was encouraged	Retrospective case-matched database study	12
				Morbidly obese patients	
				T2DM variables = HbA_1C_, med. use	
				IRB, no Reg.	
Leslie et al. [53]	USA	RYGB	Conventional medical management	Prospective study of surgical patients matched with nonsurgical database controls	24
				Morbidly obese patients	
				T2DM variables = HbA_1C_, med. use	
				IRB, no reg.	
Schauer et al. [54]	USA	RYGB (50.0 %) LSG (50.0 %)	Intensive medical therapy with lifestyle counseling by diabetes educator, weight management, encouraged to join Weight Watchers; clinic visits every 3 months	RCT, nonblinded	12
				Obese patients	
				T2DM variables = FPG, HbA_1C_, HOMA	
				IRB, IC	
				Reg. NCT00432809	
Ikramuddin et al. [55]	USA	RYGB + intensive medical management	Medical management including lifestyle modification (diet, exercise) for maximal weight loss and medication optimization	RCT, nonblinded, multicenter trial	12
				Mildly obese and obese patients	
				T2DM variables = FPG, HbA_1C_	
				IRB, IC	
				Reg. NCT00641251	

^a^Country of first author’s affiliation

*RCT* randomized controlled trial, *nRCT* nonrandomized controlled trial, *HOMA* homeostasis model assessment, *IR* insulin resistance, *IRB* institutional review and/or ethics board approval obtained, *IC* informed consent obtained, *Reg*. clinical trial registration number or governmental grant number, *BPD* biliopancreatic diversion, *RYGB* Roux-en-Y gastric bypass, *VBG* vertical banded gastroplasty, *LAGB* laparoscopic adjustable gastric banding, *LSG* laparoscopic sleeve gastrectomy, *DS* duodenal switch

Bariatric surgical procedures employed were well-accepted, frequently performed operations [57]: Roux-en-Y gastric bypass (11; 69.0 %), laparoscopic adjustable gastric banding (LAGB; 5, 25.0 %), biliopancreatic diversion (BPD; 3, 18.8 %), laparoscopic sleeve gastrectomy (LSG; 3, 18.8 %), vertical banded gastroplasty (VBG; 1, 6.3 %), and duodenal switch (DS; 1, 6.3 %). Conventional therapies ranged from intensive weight loss programs with initial VLCDs graduating to moderate calorie intake combined with lifestyle modification training and diabetologist-managed T2DM treatment; to those with structured, rehabilitative inpatient programs; some that included routine medical management of T2DM and self-monitored weight-loss and exercise plans; to a few programs with no educational plan or dietary supervision.

### Preliminary Analysis of RCTs vs OSs

Analysis of variance using pooled summary data indicated no statistically significant baseline differences between RCT surgery, RCT conventional, OS surgery, and OS conventional patient groups with respect to age ((mean [SE]) (45.8 [2.0], 47.1 [1.4], 46.6 [2.0], 49.7 [1.4]), respectively; *F*(3, 6,127) = 0.83, *p* = 0.50), BMI ((37.5 [1.7], 42.5 [1.2], 37.2 [1.8], 40.3 [1.2]); *F*(3, 6,127) = 1.23, *p* = 0.30, HbA_1C_ (8.9 [0.6], 7.6 [0.4], 7.8 [0.6], 7.1 [0.4]); *F*(3, 1,517) = 1.84, *p* = 0.14), and FPG (163.0 [8.7], 136.3 [7.9], 156.1 [11.7], 134.0 [10.43]); *F*(3, 4,926) = 0.52 (*p* = 0.67). In light of these findings, data were integrated from RCTs and OSs to make direct comparisons between bariatric surgery and conventional therapy groups at baseline.

### Patient Characteristics and Baseline Clinical Profile

The total number of patients in the included studies was 6,131; 3,076underwent bariatric surgery and 3,055 underwent conventional treatment. The mean age of patients included in this meta-analytic research was 47.8 years, ranging from 35.8 to 62.0 years. Relative to the conventional treatment group (CTG), the bariatric surgery group (BSG) was slightly younger (47.0 years [95 % CI, 45.3, 48.7] vs 48.6 years [46.6, 50.7]; pooled SMD = −0.23 [−0.28, −0.18], *p* < 0.05; *I* ^2^ = 0.0 %), and was comprised of a somewhat greater percentage of females (72.0 vs 69.0 %, *p* < 0.01). Overall baseline comparability statistics for select clinical variables (i.e., BMI [mean data available on 16 studies/100 %], HbA_1C_ [12/75 %], and FPG [11/69 %]) are presented in Table 2. The summary statistics indicated that, on average and relative to the CTG, BSG patients had a higher mean baseline BMI (40.9 kg/m^2^ [38.5, 43.3] vs 39.4 kg/m^2^ [37.3, 41.6]; pooled SMD = 0.33 [0.16, 0.51], *p* < 0.001; *I* ^2^ = 84.0 %), a higher HbA_1C_ level (8.0 % [7.1, 9.0] vs 7.7 % [6.8, 8.5]; pooled SMD = 0.39 [0.12, 0.67], *p* < 0.01; *I* ^2^ = 84.0 %), and a higher FPG level (150.3 mg/dL [135.7, 164.9] vs 143.1 mg/dL [129.8, 156.3]; pooled SMD = 0.15 [0.02, 0.28], *p* < 0.05; *I* ^2^ = 84.0 %). These data suggest that the BSG and the CTG were fairly well-matched at baseline along variables relevant to the study of T2DM remission. The mean SMD characterizing baseline differences was 0.28 (0.15–0.39), a value considered to represent a “small” statistical and clinical mean difference between groups and an approximate distribution overlap of 80.0–85.0 %.

**Table 2:** Characteristics of comparative patient groups

Study	*N* at baseline		Body mass index, kg/m^2^			HbA_1C_, %			Fasting plasma glucose, mg/dL		
			Mean (SE^a^)			Mean (SE^a^)			Mean (SE^a^)		
	Bari.	Conv.	Bari.	Conv.	*p* value^a^	Bari.	Conv.	*p* value^a^	Bari.	Conv.	*p* value^a^
Sjöström et al. [40]	1,845	1,660	42.3 (0.10)	40.0 (0.11)	<0.001	–	–	–	97.3 (0.88)	93.7 (0.84)	<0.01
O’Brien et al. [41]	40	40	33.7 (0.29)	33.5 (0.22)	NS (0.58)	–	–	–	95.4 (5.41)	90.1 (1.71)	NS (0.35)
Dixon et al. [42]	30	30	37.0 (0.49)	37.2 (0.46)	NS (0.77)	7.8 (0.22)	7.6 (0.26)	NS (0.55)	156.7 (7.03)	158.6 (8.89)	NS (0.87)
Hofsø et al. [43]	76	63	46.7 (0.65)	43.3 (0.63)	<0.001	7.1 (0.15)	5.8 (0.15)	<0.001	122.5 (4.76)	115.3 (3.86)	NS (0.25)
Adams et al. [44]	294	522	47.9 (0.47)	45.0 (0.39)	<0.001	5.7 (0.05)	5.6 (0.06)	NS (0.54)	96.9 (1.15)	96.3 (1.30)	NS (0.76)
Serrot et al. [45]^c^	17	17	34.6 (1.38)	34.0 (1.29)	NS (0.75)	8.2 (0.32)	7.0 (0.28)	<0.005	–	–	–
Martins et al. [46]	50	129	45.2 (0.76)	45.6 (0.51)	NS (0.67)	–	–	–	–	–	–
Iaconelli et al. [47]	22	28	50.5 (1.81)	51.5 (1.17)	NS (0.63)	8.0 (0.28)	8.0 (0.22)	NS (0.99)	156.7 (10.7)	156.7 (7.00)	NS (0.99)
Scopinaro et al. [48]	30	38	30.6 (0.53)	30.2 (0.57)	NS (0.62)	9.3 (0.27)	8.3 (0.13)	<0.001	220.0 (12.6)	171.0 (6.16)	<0.001
Leonetti et al. [49]	30	30	41.3 (1.10)	39.0 (1.00)	NS (0.13)	7.9 (0.38)	8.1 (0.31)	NS (0.69)	166.0 (12.4)	183.0 (11.6)	NS (0.32)
Mingrone et al. [50]	40	20	45.0 (1.03)	45.6 (1.39)	NS (0.73)	8.7 (0.25)	8.5 (0.27)	NS (0.62)	173.4 (9.68)	179.0 (13.8)	NS (0.74)
Heo et al. [51]	261	224	39.0 (0.38)	34.3 (0.25)	<0.001	–	–	–	–	–	–
Dorman et al. [52]	29	29	42.4 (0.56)	40.2 (0.80)	<0.05	7.2 (0.20)	7.2 (0.22)	NS (0.99)	–	–	–
Leslie et al. [53]	152	115	47.4 (0.54)	40.7 (0.47)	<0.001	7.6 (0.10)	7.2 (0.10)	<0.01	–	–	–
Schauer et al. [54]	100	50	36.6 (0.36)	36.3 (0.42)	NS (0.61)	9.4 (0.16)	8.9 (0.20)	NS (0.06)	178.5 (5.27)^c^	155.0 (6.29)^c^	<0.01
Ikramuddin et al. [55]	60	60	34.9 (0.39)	34.3 (0.40)	NS (0.28)	9.6 (0.13)	9.6 (0.16)	NS (0.99)	222.0 (9.94)	207.0 (7.36)	NS (0.23)
Total	3,076	3,055	–	–	–	–	–	–	–	–	–
IV Weighted mean	–	–	40.9 (1.23)	39.4 (1.10)		8.0 (0.50)	7.7 (0.45)		150.3 (7.5)	143.1 (6.77)	
(95 % CI)			(38.5, 43.3)	(37.3, 41.6)		(7.1, 9.0)	(6.8, 8.5)		(135.7, 164.9)	(129.8, 156.3)	
Pooled SMD^b^				0.33 (0.09)		–	0.39 (0.14)			0.15 (0.07)	
(95 % CI)	–	–	–	(0.16, 0.51)			(0.12, 0.67)		–	(0.02, 0.28)	
*p* value				<0.001			<0.01			<0.05	

^a^Standard errors and *p* values are representative of meta-analytic data/results

^b^Cohen’s *d* (SMD) is interpreted as: 0.2 = small effect size (or small standardized mean difference), 0.5 = medium effect size, 0.8 = large effect size

^c^Median value used instead of mean for summary calculations

### Assessment of Within-Group Change in Clinical Markers After Treatment

Table 3 presents meta-analytic data summarizing mean baseline (pretreatment), mean follow-up (post-treatment), and mean change values in BMI, HbA_1C_, and FPG for each of the studies within the two treatment groups (i.e., BSG and CTG). Pooled estimates of overall means and mean change (WMD) are also provided. The weighted mean baseline BMI for the BSG was 40.9 kg/m^2^ (95 % CI, 38.5, 43.3), with a follow-up weighted mean BMI of 29.4 kg/m^2^ (27.8, 30.9). The WMD in BMI for BSG patients was 11.4 kg/m^2^ ([95 % CI, 10.0, 12.9], statistical significance of overall effect: *p* < 0.001, *I* ^2^ = 95.0 %). In contrast, the weighted mean baseline BMI for the CTG was 39.4 kg/m^2^ (37.3, 41.6), with a follow-up weighted mean BMI of 37.8 kg/m^2^ (35.6, 39.9). The WMD for all patients undergoing some form of conventional therapy was 1.6 kg/m^2^ ([0.7, 2.6], *p* < 0.01, *I* ^2^ = 86.5 %).

**Table 3:** Pre/postcomparative body mass index, HbA_1C_, and fasting plasma glucose outcomes

Study	Bariatric procedure				Conventional treatment			
	Pre	Post	Change (95 % CI)	*p* value^a^	Pre	Post	Change (95 % CI)	*p* value^a^
Body mass index, kg/m^2^ mean (SE^a^)								
Sjöström et al. [40]	42.3 (0.10)	32.4 (0.12)	9.9 (9.6, 10.2)	<0.001	40.0 (0.11)	40.04 (0.13)	−0.04 (−0.38,0.30)	NS (0.82)
O’Brien et al. [41]	33.7 (0.29)	26.4 (0.58)	7.3 (6.0, 8.6)	<0.001	33.5 (0.22)	31.5 (0.91)	2.0 (0.36, 3.64)	<0.05
Dixon et al. [42]	37.0 (0.49)	29.5 (0.67)	7.5 (5.9, 9.1)	<0.001	37.2 (0.46)	36.6 (0.92)	0.6 (−1.4, 2.6)	NS (0.56)
Hofsø et al. [43]	46.7 (0.65)	32.7 (0.57)	14.0 (12.3, 15.7)	<0.001	43.3 (0.63)	39.6 (0.68)	3.7 (1.9, 5.5)	<0.001
Adams et al. [44]	47.9 (0.47)	32.2 (0.47)	15.5 (14.2,16.8)	<0.001	45.0 (0.39)	44.6 (0.39)	0.4 (−0.69, 1.5)	NS (0.47)
Serrot et al. [45]	34.6 (1.38)	25.8 (1.21)	8.8 (5.2, 12.4)	<0.001	34.0 (1.29)	34.3 (1.32)	−0.3 (−3.9, 3.3)	NS (0.87)
Martins et al. [46]	45.2 (0.76)	31.1 (0.71)	14.1 (12.1, 16.1)	<0.001	45.6 (0.51)	40.7 (0.48)	4.9 (3.5, 6.3)	<0.001
Iaconelli et al. [47]	50.5 (1.81)	34.6 (1.07)	15.9 (11.8, 20.0)	<0.001	51.5 (1.17)	43.6 (1.02)	7.9 (4.9, 10.9)	<0.001
Scopinaro et al. [48]	30.6 (0.53)	25.3 (0.42)	5.3 (4.0, 6.6)	<0.001	30.2 (0.57)	30.2 (0.58)	0.0 (−1.6, 1.6)	NS (0.99)
Leonetti et al. [49]	41.3 (1.10)	28.3 (0.99)	13.0 (10.1, 15.9)	<0.001	39.0 (1.00)	39.8 (0.91)	−0.8 (−3.5, 1.9)	NS (0.56)
Mingrone et al. [50]	45.0 (1.03)	29.3 (0.62)	15.8 (13.4, 18.1)	<0.001	45.6 (1.39)	43.1 (1.51)	2.5 (−1.5, 6.5)	NS (0.22)
Heo et al. [51]	39.0 (0.38)	30.2 (0.75)	8.8 (6.9, 10.7)	<0.001	34.3 (0.25)	32.0 (0.89)	2.3 (0.89, 3.7)	<0.05
Dorman et al. [52]	42.4 (0.56)	27.6 (0.52)	14.8 (13.3, 16.3)	<0.001	40.2 (0.80)	40.6 (0.84)	−0.4 (−2.7, 1.9)	NS (0.73)
Leslie et al. [53]	47.4 (0.54)	32.4 (0.53)	15.0 (13.5, 16.5)	<0.001	40.7 (0.47)	40.8 (0.47)	−0.1 (−1.4, 1.2)	NS (0.88)
Schauer et al. [54]	36.6 (0.36)	27.0 (0.37)	9.6 (8.6, 10.6)	<0.001	36.3 (0.42)	34.4 (0.79)	1.9 (0.23, 3.57)	<0.05
Ikramuddin et al. [55]	34.9 (0.39)	25.8 (0.46)	9.1 (7.9, 10.3)	<0.001	34.3 (0.40)	31.6 (0.49)	2.7 (1.5, 3.9)	<0.001
Weighted mean	40.9 (1.23)	29.4 (0.81)	11.4 (0.73) WMD		39.4 (1.10)	37.8 (1.08)	1.6 (0.49) WMD	
(95 % CI)	(38.5, 43.3)	(27.8, 30.9)	(10.0, 12.9)	<0.001^a^	(37.3, 41.6)	(35.6, 39.9)	(0.7, 2.6)	<0.001^a^
HbA_1C_, % mean (SE^a^)								
Dixon et al. [42]	7.8 (0.22)	6.0 (0.15)	1.8 (1.3, 2.3)	<0.001	7.6 (0.26)	7.2 (0.25)	0.4 (−0.3, 1.1)	NS (0.28)
Hofsø et al. [43]	7.1 (0.15)	6.6 (0.12)	0.5 (0.1, 0.9)	<0.05	5.8 (0.15)	6.3 (0.14)	−0.5 (−0.9, −0.1)	<0.05
Adams et al. [44]	5.7 (0.05)	5.6 (0.05)	0.1 (−0.04, 0.24)	NS (0.16)	5.6 (0.06)	5.8 (0.06)	−0.2 (−0.4, −0.1)	<0.05
Serrot et al. [45]	8.2 (0.32)	6.1 (0.25)	2.1 (1.3, 2.9)	<0.001	7.0 (0.28)	7.1 (0.27)	−0.1 (−0.9, 0.7)	NS (0.80)
Iaconelli et al. [47]	8.0 (0.28)	5.2 (0.22)	2.8 (2.1, 3.5)	<0.001	8.0 (0.22)	7.2 (0.21)	0.8 (0.2, 1.4)	<0.05
Scopinaro et al. [48]	9.3 (0.27)	6.5 (0.15)	2.8 (2.2, 3.4)	<0.001	8.3 (0.13)	7.7 (0.11)	0.6 (0.3, 0.9)	<0.005
Leonetti et al. [49]	7.9 (0.38)	6.0 (0.27)	1.9 (1.0, 2.8)	<0.001	8.1 (0.31)	7.1 (0.24)	1.0 (0.2, 1.8)	<0.05
Mingrone et al. [50]	8.7 (0.25)	5.7 (0.16)	3.0 (2.4, 3.6)	<0.001	8.5 (0.27)	7.7 (0.14)	0.8 (0.2, 1.4)	<0.05
Dorman et al. [52]	7.2 (0.20)	5.9 (0.19)	1.3 (0.8, 1.8)	<0.001	7.2 (0.22)	7.3 (0.26)	−0.1 (−0.8, 0.6)	NS (0.77)
Leslie et al. [53]	7.6 (0.10)	6.4 (0.10)	1.2 (0.9, 1.5)	<0.001	7.2 (0.10)	7.2 (0.10)	0.0 (−0.3, 0.3)	NS (0.99)
Schauer et al. [54]	9.4 (0.16)	6.5 (0.10)	2.9 (2.5, 3.3)	<0.001	8.9 (0.20)	7.5 (0.28)	1.4 (0.7, 2.1)	<0.001
Ikramuddin et al. [55]	9.6 (0.13)	6.3 (0.12)	3.3 (3.0, 3.7)	<0.001	9.6 (0.16)	7.8 (0.20)	1.8 (1.3, 2.3)	<0.05
Weighted mean	8.0 (0.50)	6.1 (0.15)	2.0 (.40) WMD		7.7 (0.45)	7.2 (0.27)	0.47 (0.19) WMD	
**(**95 % CI	(7.1, 9.0)	(5.8, 6.4)	(1.2, 2.8)	<0.001^a^	(6.8, 8.5)	(6.6, 7.7)	(0.1, 0.9)	<0.05^a^
Fasting plasma glucose, mg/dL mean (SE^a^)								
Sjöström et al. [40]	97.3 (0.88)	84.1 (0.7)	13.2 (11.0, 15.4)	<0.001	93.7 (0.84)	98.5 (0.8)	−4.8 (−7.1, −2.5)	<0.001
O’Brien et al. [41]	95.4 (5.4)	88.4 (5.3)	7.0 (−7.8, 21.8)	NS (0.35)	90.1 (1.7)	89.8 (7.6)	0.3 (−13.3, 13.9)	NS (0.99)
Dixon et al. [42]	156.7 (7.0)	105.6 (5.5)	51.1 (33.6, 68.6)	<0.001	158.6 (8.9)	139.6 (7.0)	19.0 (−3.1, 41.1)	NS (0.09)
Hofsø et al. [43]	122.5 (4.8)	88.3 (3.3)	34.2 (22.8, 45.6)	<0.001	115.3 (3.9)	100.9 (4.3)	14.4 (3.1, 25.7)	<0.05
Adams et al. [44]	96.9 (1.2)	82.0 (1.2)	14.9 (11.7, 18.0)	<0.001	96.3 (1.3)	97.3 (1.3)	−1.0 (−4.6, 2.6)	NS (0.58)
Iaconelli et al. [47]	156.7 (10.7)	75.7 (6.2)	81.0 (56.8, 105.2)	<0.001	156.7 (7.0)	126.1 (6.4)	30.6 (11.9, 49.2)	<0.01
Scopinaro et al. [48]	220.0 (12.6)	149.0 (7.5)	71.0 (42.3, 99.7)	<0.001	171.0 (6.2)	151.0 (4.5)	20.0 (5.0, 35.0)	<0.01
Leonetti et al. [49]	166.0 (12.4)	97.0 (5.3)	69.0 (42.5, 95.5)	<0.001	183.0 (11.6)	150.0 (8.8)	33.0 (4.5, 61.5)	<0.05
Mingrone et al. [50]	173.4 (9.7)	86.3 (5.5)	87.1 (65.0,109.2)	<0.001	179.0 (13.8)	141.1 (7.1)	37.9 (6.5, 69.3)	<0.05
Schauer et al. [54]^b^	178.5 (5.3)	98.0 (3.3)	80.5 (68.3, 92.7)	<0.001	155.0 (6.3)	120.0 (6.6)	35.0 (17.0, 53.0)	<0.001
Ikramuddin et al. [55]	222.0 (9.9)	111.0 (4.5)	111.0 (89.2,132.8)	<0.001	207.0 (7.4)	153.0 (7.8)	54.0 (33.0, 75.0)	<0.001
Weighted mean	150.3 (7.5)	95.3 (3.1)	53.3 (6.8) WMD		143.1 (6.8)	123.2 (5.1)	17.4 (4.4) WMD	
(95 % CI)	(135.7, 164.9)	(89.3, 101.3)	(40.0, 66.7)	<0.001^a^	(129.8, 156.3)	(113.3, 133.1)	(8.8, 26.0)	<0.001^a^

*WMD* weighted mean difference

^a^Standard errors and *p* values are representative of meta-analytic results

^b^Median value used instead of mean for summary calculations

**Fig. 2:**
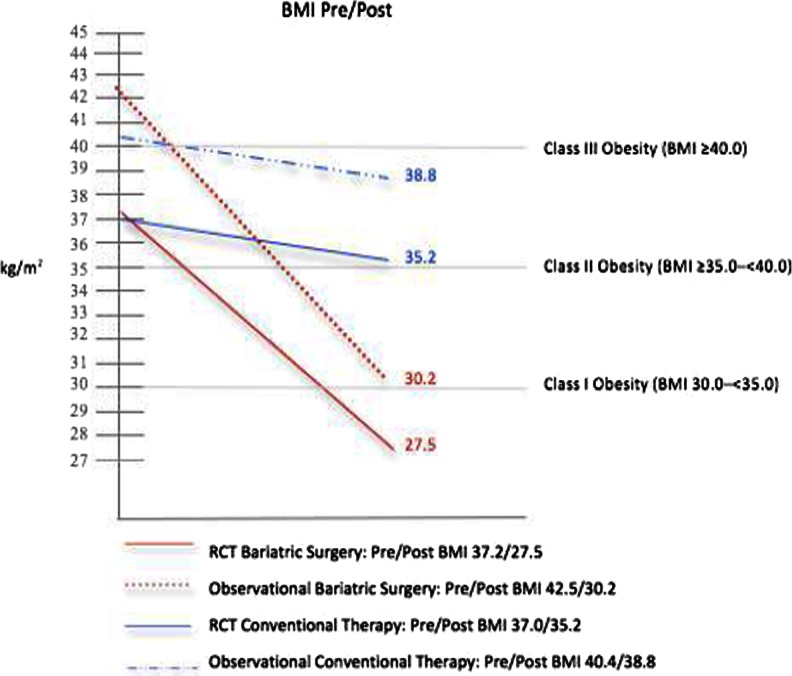
Mean body mass index (BMI) reduction in bariatric surgery patients and conventional therapy patients by study design (randomized controlled trial vs observational)

**Fig. 3:**
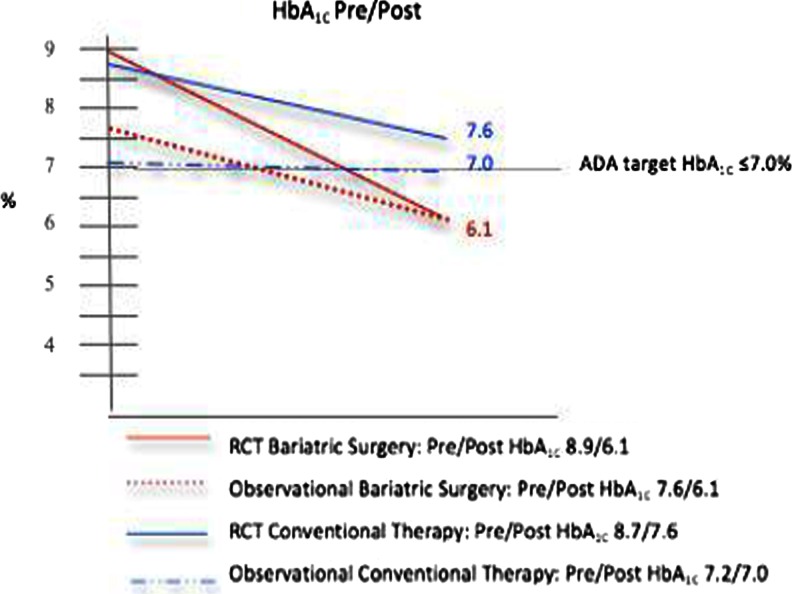
Mean glycated hemoglobin (HbA_1C_) reduction in bariatric surgery patients and conventional therapy patients by study design (randomized controlled trial vs observational)

Within-group mean changes in HbA_1C_ and FPG tended to follow the pattern of change observed in BMI. At baseline, the BSG had a weighted mean HbA_1C_ of 8.0 % (7.1, 9.0), with a follow-up of 6.1 % (5.8, 6.4), a reduction that represented an overall WMD of 2.0 % ([1.2, 2.8], *p* < 0.001, *I* ^2^ = 86.5 %) for BSG patients. The CTG was found to have a weighted mean baseline HbA_1C_ of 7.7 % (6.8, 8.5), with a follow-up of 7.2 % (6.6, 7.7). The WMD in HbA_1C_ levels following conventional therapy was 0.47 % ([0.1, 0.9], *p* < 0.05; *I* ^2^ = 90.1 %). Similarly, the BSG had a baseline weighted mean FPG of 150.3 mg/dL (135.7, 164.9), with a follow-up of 95.3 mg/dL (89.3, 101.3). The WMD was 53.3 mg/dL ([40.0, 66.7], *p* < 0.001; *I* ^2^ = 96.8 %). Finally, baseline weighted mean FPG for the CTG was 143.1 mg/dL (129.8, 156.3), with a follow-up of 123.2 mg/dL (113.3, 133.1). The WMD in FPG levels for conventional therapy patients was 17.4 mg/dL ([8.8, 26.0], *p* < 0.001; *I* ^2^ = 89.2 %). Figures 2 and 3 depict trend lines characterizing the relative changes over time in BMI and HbA_1C_ levels for BSG and CTG groups stratified by study design.

### Assessment of Between-Group Differences in Clinical Markers After Treatment

The WMD comparing BSG and CTG on follow-up BMI for combined OS data (*k* = 11; *n* = 5,257) was −8.5 kg/m^2^ ([−10.2, −6.9], *p* < 0.001; *I* ^2^ = 93.0 %); whereas, the corresponding WMD for combined RCT data (*k* = 5; *n* = 440) was −7.7 kg/m^2^ ([−10.1, −5.3], *p* < 0.001; *I* ^2^ = 87.5 %), with a high degree of 95 % CI overlap. No significant heterogeneity (*Q p* value = 0.573) was found between the OSs’ summary estimate and the RCTs’ summary estimate with respect to the magnitude and direction of treatment effect on BMI, with the surgery group favored in both study designs.

The WMD comparing BSG vs CTG on follow-up HBA_1C_, for combined OS data (*k* = 8; *n* = 1,131) was −0.89 % ([−1.3, −0.45], *p* < 0.001; *I* ^2^ = 91.7 %); whereas, the corresponding WMD for combined RCT data (*k* = 4; *n* = 370) was −1.43 % ([−2.1, −0.81], *p* < 0.001; *I* ^2^ = 66.2 %), with significant 95 % CI overlap. No significant heterogeneity (*Q p* value = 0.16) was found between the OSs’ summary estimate and the RCTs’ summary estimate with respect to the magnitude and direction of treatment effect on HBA_1C_, with the surgery group favored in both study designs.

The WMD comparing BSG vs CTG on follow-up FPG, for combined OS data (*k* = 6; *n* = 4,460) was −20.9 mg/dL ([−29.3, −12.5]; *p* < 0.001; *I* ^2^ = 84.3 %); whereas, the corresponding WMD for combined RCT studies (*k* = 5; *n* = 440) was −30.1 mg/dL ([−40.8, −19.5], *p* < 0.001; *I* ^2^ = 80.5 %), with a significant 95 % CI overlap. No significant heterogeneity (*Q p* value = 0.18) was found between the OSs’ summary estimate and the RCTs’ summary estimate with respect to the magnitude and direction of treatment effect on FPG, with the surgery group favored in both study designs.

**Fig. 4:**
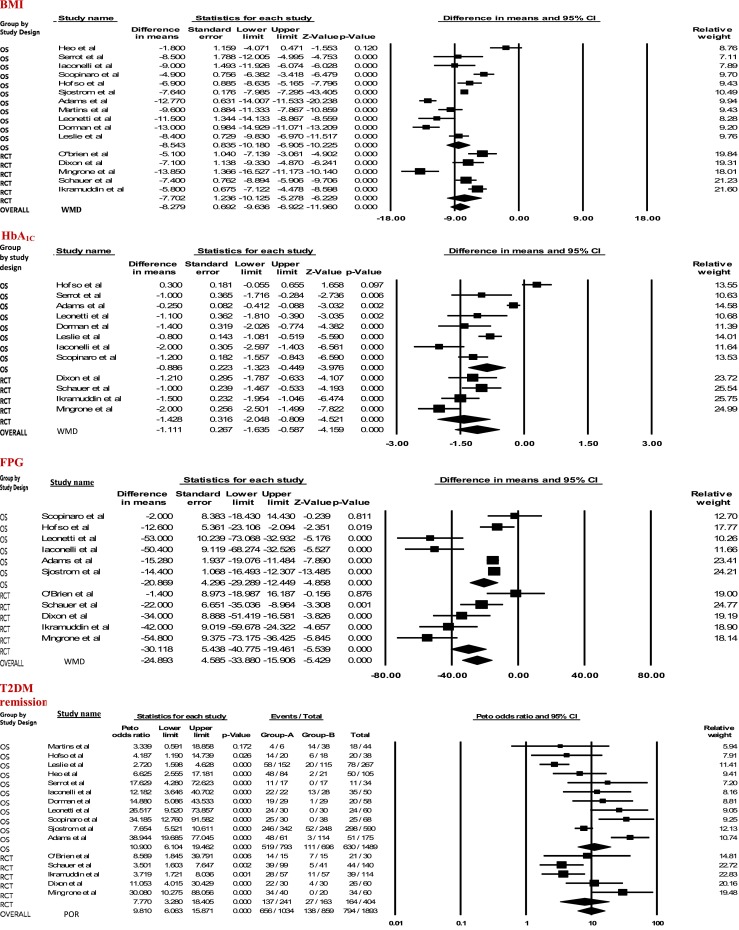
The first three tables and corresponding forest plots summarize meta-analyses of the relative effects of bariatric surgery vs conventional therapy on body mass index (BMI), glycated hemoglobin (HbA_1C_), and fasting plasma glucose (FPG). Each study contributing to a particular meta-analysis is represented by a *single darkened square* contained on the forest plot; the size of the square being proportional to the amount of weight the study was given during the calculation of the pooled summary estimate. The pooled estimate in the first three analyses is expressed as the weighted mean difference (WMD) and is represented by the *diamond shape* at the base of each forest plot. Two additional diamonds in each forest plot represent independent summary estimates for observational studies and randomized controlled trials. Negative WMD values indicate a treatment effect favoring surgical intervention. The fourth table (and forest plot) represents an analysis of the relative effects of surgery vs conventional therapy on T2DM remission. In this case, the summary estimate of effect is given by the pooled odds ratio (POR). Results favor surgery over conventional therapy when odds ratio values are greater than one

In summary, independent treatment effect sizes for both OSs and RCTs were sufficiently concordant to permit estimation of an overall effect for each analysis presented in Fig. 4. The first three meta-analytic results (BMI, HbA_1C_, and FPG) provide individual study mean follow-up differences and SEs, as well as independent summary estimates for OSs and RCTs, and the overall WMD for combined included studies. Negative mean difference values indicate a treatment effect favoring surgical intervention; OR results favored surgery over conventional therapy where values and plotting points comprising the forest chart are >1.0.

The overall (*k* = 16; *n* = 5,697) between-group WMD characterizing BMI outcomes was −8.3 kg/m^2^ ([−9.6, −6.9], *p* < 0.001; *I* ^2^ = 91.8 %), favoring the surgery group. The corresponding pooled SMD was −1.62 ([−1.8, −1.4]; *p* < 0.001; *I* ^2^ = 90.1 %); adjusted effect size, −1.95 [−2.15, −1.76]. Analysis of BMI using studies with complete data (*k* = 6 studies, no imputation) yielded a WMD of −9.5 kg/m^2^ ([−12.3, −6.6], *p* < 0.001; *I* ^2^ = 93.6 %) and corresponding pooled SMD of −2.1([−2.7, −1.6], *p* < 0.001; *I* ^2^ = 85.2 %).

WMD calculations summarizing between-group comparisons along the HbA_1C_ and FPG variable outcomes yielded similar results to those found for BMI. The overall (*k* = 12; *n* = 1,501) WMD for HBA_1C_ outcomes was −1.1 % ([−1.6, −0.6], *p* < 0.001; *I* ^2^ = 91.9 %), again, favoring the surgery group. The corresponding pooled SMD was −1.0 ([−1.4, −0.6], *p* < 0.001; *I* ^2^ = 89.2 %); adjusted effect size, −1.39 ([−1.72, −1.01]). Analysis of HbA_1C_ using studies with complete data (*k* = 8 studies, no imputation) yielded a WMD of −1.3 % ([−1.54, −0.98], *p* < 0.001; *I* ^2^ = 65.6 %) and pooled SMD of −1.13 ([−1.4, −0.8], *p* < 0.001; *I* ^2^ = 73.2 %).

Finally, the overall (*k* = 11; *n* = 4,900) between-group FPG WMD was −24.9 mg/dL ([−33.9, −15.9]; *p* < 0.001; *I* ^2^ = 84.8 %), favoring the surgery group. The pooled SMD was −0.71 ([−0.92, −0.50], *p* < 0.001; *I* ^2^ = 80.5 %; adjusted effect size, −0.86 [−1.05, −0.67]). Analysis of FPG using studies with complete data (*k* = 5 studies, no imputation) yielded a WMD of −36.8 mg/dL ([−56.3, −17.3], *p* < 0.001; *I* ^2^ = 83.1 %) and pooled SMD of −0.96 ([−1.5, −0.5], *p* < 0.001; *I* ^2^ = 79.9 %).

### Effects of Bariatric Surgery vs Conventional Therapy on %EWL and T2DM

**Fig. 5:**
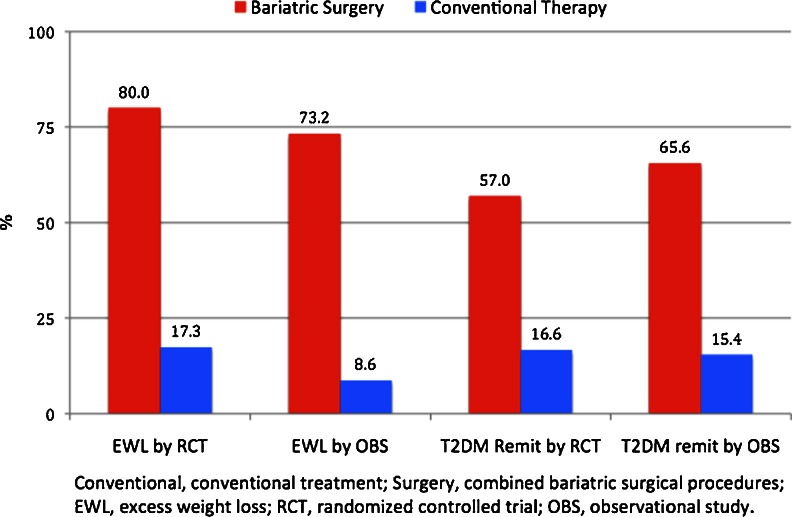
Mean percent excess weight loss (%EWL) in bariatric surgery patients and conventional therapy patients by study design type (randomized controlled trial vs observational)

Overall EWL means for the BSG and the CTG groups were 75.3 % (57.2–94.6) and 11.3 % (−5.7–29.8), respectively; overall T2DM remission rates were 63.5 % (38.2–100.0) and 15.6 % (0.0–46.7) (*p* < 0.001; Table 4). Figure 5 presents a further breakdown of %EWL and T2DM remission by treatment group and study design. Bariatric surgery patients enrolled in RCT designs reported the highest mean EWL (80.0 %); bariatric surgery patients enrolled in OSs had the highest T2DM remission rate (65.6 %).

**Table 4:** Excess weight loss and diabetes remission

Study	%EWL^a^		T2DM remission rate		
	Mean		% (*N*)		
	Bariatric	Conventional	Bariatric	Conventional	*p* value^b^
Sjöström et al. [40]	57.2	−0.3	72.0 (342)	21.0 (248)	<0.001
O’Brien et al. [41]	83.9	23.5	93.0 (15)	46.7 (15)	<0.01
Dixon et al. [42]	62.5	4.9	73.0 (30)	13.0 (30)	<0.001
Hofsø et al. [43]	64.5	20.2	79.0 (14)	0.0 (6)	<0.005
Adams et al. [44]	69.6	1.9	78.7 (61)	2.6 (114)	<0.001
Serrot et al. [45]	91.7	3.3	64.7 (17)	0.0 (17)	<0.001
Martins et al. [46]	69.8	23.8	67.0 (6)	36.8 (38)	NS (0.17)
Iaconelli et al. [47]	62.4	29.8	100.0 (22)	45.0 (28)	<0.001
Scopinaro et al. [48]^c^	94.6	0.0	83.0 (30)	0.0 (38)	<0.001
Leonetti et al. [49]	79.8	−5.7	80.0 (30)	0.0 (30)	<0.001
Mingrone et al. [50]	79.0	12.1	85.0 (40)	0.0 (20)	<0.001
Heo et al. [51]	62.9	24.7	57.1 (84)	9.5 (21)	<0.001
Dorman et al. [52]	85.1	−2.6	65.0 (29)	3.4 (29)	<0.001
Leslie et al. [53]	67.0	−0.6	38.2 (152)	17.4 (115)	<0.001
Schauer et al. [54]^d^	82.8	16.8	39.4 (99)	12.0 (41)	<0.005
Ikramuddin et al. [55]	91.9	29.0	49.0 (57)	19.0 (57)	<0.001
Overall	75.3	11.3	63.5 (1,028)	15.6 (847)	<0.001
(Range)	(57.2–94.6)	(−5.7–29.8)	(38.2–100.0)	(0.0–46.7)	

Negative values in the %EWL column denote mean weight gain

*EWL* excess weight loss, *T*2*DM* type 2 diabetes mellitus

^a^Standardized calculation using BMI 25 as ideal weight constant

^b^ *Z* test for two population proportions

^c^Patients with controlled diabetes following treatment included in remission rate calculation

^d^Patients recovering from metabolic syndrome following treatment included in remission rate calculation

T2DM remission event data, PORs, and 95 % CIs describing the effects of surgery vs conventional therapy on T2DM remission are presented in the final forest plot of Fig. 4. Independent summary estimates were calculated for RCTs and OSs using the Peto method. The POR and 95 % CI for combined OS remission event data (*k* = 11; *n* = 1,489) was 10.9 ([6.1, 19.5], *p* < 0.001; *I* ^2^ = 81.6 %); whereas, the corresponding POR and 95 % CI for combined RCT remission event data (*k* = 5; *n* = 404) was 7.8 ([3.3, 18.4], *p* < 0.001; *I* ^2^ = 70.2 %), with a high degree of 95 % CI overlap. No significant heterogeneity (*Q p* value = 0.52) was found between the OSs’ estimate and the RCTs’ estimate with respect to the magnitude and direction of treatment effect on T2DM remission, with the surgery group favored in both study designs. Evaluation by *z* score also indicated no statistically significant difference between OS and RCT summary estimates (*z* = −0.57; *p* = 0.57; a *z* score ≤−1.96 or ≥1.96 would indicate a statistically significant difference at the 0.05 level). Thus, estimates were sufficiently concordant to calculate an overall effect (i.e., combining data from RCTs with OSs). As shown in Fig. 4, the (*k* = 16; *n* = 1,893) summary POR was 9.8 ([6.1, 15.9], *p* < 0.001; *I* ^2^ = 78.4 %).

Independent PORs and 95 % CIs were also calculated using the inverse variance method as a direct comparison to Peto findings. The POR and 95 % CI for combined OS data was 18.9 ([8.1, 43.7], *p* < 0.001; *I* ^2^ = 79.9 %); whereas, the corresponding POR and 95 % CI for combined RCT data was 11.0 ([3.3, 36.3], *p* < 0.001; *I* ^2^ = 59.9 %), with significant 95 % CI overlap. No significant heterogeneity (*Q p* value = 0.47) was found between the OS and RCT summary estimates, where the surgery group was favored in both study designs. Evaluation by *z* score also indicated no statistically significant difference between the independent summary estimates (*z* = −0.60; *p* = 0.55), the inverse variance method yielded an overall summary POR of 15.8 ([7.9, 31.4], *p* < 0.001; *I* ^2^ = 75.2 %). Thus, according to the inverse variance method, the odds of T2DM remission in patients undergoing bariatric surgery were, on average, 15.8 times the odds of remission for those receiving conventional therapy. All but one of 16 studies indicated a clear statistical advantage favoring surgery.

### Sensitivity and Subgroup Analyses

Three sensitivity analyses were performed: (1) an analysis excluding trials reporting no remission events; (2) an analysis excluding studies that combined data from multiple study arms; and (3) an analysis excluding all bariatric procedures except RYGB (the most frequently performed procedure). [See Electronic Supplementary Material, Appendices 2 and 3 to read the detailed results of these subgroup analyses and an assessment of publication bias (includes Table 5 and Fig. 6).]

## Discussion

The current meta-analysis systematically identified and integrated a wide range of evidence regarding the effectiveness of bariatric surgery vs conventional therapy in promoting weight loss and T2DM remission. Results indicated that bariatric surgery demonstrated greater BMI reduction, greater reduction in HbA_1C_ and FPG, and a much greater likelihood of T2DM remission relative to patients receiving conventional therapy.

### Heterogeneity

According to the prespecified *I* ^2^ value (≥75.0 %), a majority of meta-analyses undertaken in this study were characterized by significant heterogeneity (mean *I* ^2^, 81.4 % [0–96.8]; mean RCT, *I* ^2^ = 72.0; mean OS, *I* ^2^ = 86.0). Generally, when effect estimates from individual studies rest on opposite sides of the reference line (i.e., the point of no effect), study results are, by definition, heterogeneous, and conclusions questionable. However, the meta-analytic results presented in Fig. 4 showed the vast majority of estimated effects were consistently anchored on the same side of the reference line, with 95 % CIs overlapping to a great extent. Thus, when viewed in isolation, the mean *I* ^2^ statistic may be somewhat misleading.

One source of heterogeneity was expected as a result of integration of RCT and OS data. The majority of bariatric surgery studies are observational in design, as RCTs are less feasible to conduct for ethical and economic reasons [58]. Most RCTs are conducted in high-volume “centers of excellence” according to regulated protocols that conform to national guidelines [59, 60]; therefore, RCT outcomes may differ significantly from OSs. Yet, interestingly, the current review’s comparative analysis of trends in outcome variables, stratified by study design, was highly similar (Figs. 2, 3, and 5 [see Fig. 5 in online ESM 3]). In addition, meta-analytic results indicated no significant heterogeneity between RCT and OS summary estimates quantifying the relative effects of bariatric surgery vs conventional therapy on BMI, HbA_1C_, and FPG reduction. In addition, both study designs demonstrated the superiority of bariatric surgery over conventional therapy in promoting T2DM remission. A common criticism of OSs, in general, is that they produce exaggerated effect sizes. While it is true that the POR for T2DM remission derived from OS studies was larger than that derived from RCTs (Peto, 10.9 [6.1, 19.5] vs 7.8 [3.3, 18.4]; inverse variance, 18.9 [8.1, 43.7] vs 11.0 [3.3, 36.3]), no significant heterogeneity was found between summary estimates. Further, Shrier et al., in their review of the principal elements underlying this claim, found that both study designs have strengths and weaknesses, and including OSs would increase precision appropriately, and may produce equally or more relevant and valid results [61].

### Weight

#### Conventional Treatment

To place the current meta-analytic findings in context, they should be compared to publications outside of the included study set. The current findings in relation to BMI reduction (WMD 1.6 kg/m^2^ [0.7, 2.6]) and EWL (11.3 % [−5.7–29.8]) after conventional treatment are similar to those of key systematic reviews (SRs) and meta-analyses (MAs) [62, 63]. A SR by Tsai et al., focused on weight-loss programs across the USA, saw 15.0–25.0 % excess body weight loss over 3–6 months, although fewer than 9.0 % of the patients maintained their weight loss at 12 months [62]. In a MA by Dansinger et al. of 46 trials that provided dietary counseling, BMI was reduced by 1.9 units at 1 year (comparable to the 1.6 BMI units lost by the conventional therapy group in the current meta-analysis), with a per-month BMI loss of approximately 0.1 unit between 3 and 12 months of the active counseling programs, with a BMI regain of approximately 0.02–0.03 unit per month during follow-on maintenance phases [63].

Interestingly, while excluded from the current analysis, a 2005 comparative study, by Ritt et al., of 24 LAGB vs 16 conventional therapy patients reported an atypically large EWL (54.5 %) [64], well outside the mean EWL (11.3 %) for conventional therapy patients reported in our review. Also, in a 2007 OS by Anderson et al. of 1,531 morbidly obese conventional weight-loss patients with long-term follow-up employing very intensive behavioral intervention (e.g., weight-loss camps, residential nursing programs, and closely supervised individualized outpatient programs, possibly cost prohibitive for many patients), marked weight loss was achieved up to 100 lbs over the short term; however, over 1–5-year follow-up, most patients regained 34.0–41.0 % of their lost weight [65].

#### Bariatric Surgery

The current findings for bariatric surgery in relation to BMI reduction (WMD, 11.4 kg/m^2^ [10.0, 12.9]) and EWL (75.3 % [57.2–94.6]) were similar to those of two SR/MAs of the bariatric literature by Buchwald et al. [5, 22]; aggregated weight outcomes in morbidly obese patients found 13.6 kg/m^2^ (12.9, 14.3) and 14.2 kg/m^2^ (13.2, 15.1) BMI reduction, 64.7 % (32.0–93.0) and 55.9 % (54.1, 57.8) EWL, respectively [5, 22]. Also, an SR by Gill et al. of T2DM patients who underwent sleeve gastrectomy (SG) with a mean 13 months of follow-up noted 47.0 % EWL [66].

#### Conventional Treatment vs Bariatric Surgery

A rigorous 2009 Cochrane review by Colquitt et al. of mildly and morbidly obese patients both with and without diabetes found that over the short term, bariatric surgery achieved substantially greater weight loss than medical therapy [67], as was concluded also by Maggard-Gibbons et al., of mildly obese diabetic patients in their 2013 SR [68]. Padwal et al.’s 2011 SR/MA of 31 RCTs, in which bariatric surgery was compared with other bariatric surgery controls or standard care (*n* = 2,619; BMI 42.0–58.0) using network analysis, found conventional treatment significantly less effective than surgery over the short and intermediate terms. Padwal et al. also found an average BMI WMD of 8.35 kg/m^2^ (2.4–11.4) in their subset comparative study of all surgery vs conventional therapy [69]. The current review’s between-group BMI WMD was nearly identical (8.3 kg/m^2^ [7.0, 9.6]) to the results of Padwal et al., which would be expected since the current authors grouped procedures for analysis. In addition, the pooled adjusted SMD (corresponding to the BMI WMD of 8.3 kg/m^2^) was 1.95 (1.76, 2.15), indicating that the average surgery patient experienced a BMI outcome superior to that of 97.1 % of the conventional therapy group. While both treatment groups in the current review can be said to have experienced a statistically significant within-group BMI change, only the bariatric surgery patients experienced a clinically meaningful weight reduction, as demonstrated by a mean shift downward by as much as two obesity classifications.

### Diabetes

#### Conventional Treatment

The long-running Look AHEAD study of weight loss by conventional treatment (*n* = 5,145 overweight adults with T2DM) found an association between intensive weight-loss intervention (ILI) and a reduction in T2DM markers: Mean HbA_1C_ dropped from 7.25 to 6.61 % (*p* < 0.00). Although the degree of success in ILI diabetes reduction experienced in the Look AHEAD study resulted in a seemingly greater outcome than that of the current meta-analysis, the Look AHEAD ILI group started at a lower HbA_1C_ than the conventional group in the current analysis, and their overall HbA_1C_ reduction was proportionally similar. By this measure, Look AHEAD results suggest that this form of therapy might be appropriate to those patients hovering around the 7.0 % HbA_1C_ mark at baseline. Look AHEAD FPG outcomes for the ILI group dropped from 151.9 to 130.4 mg/dL, and were essentially identical to those of the conventional group in the current analysis [70]; the FPG reduction of the current analysis compared favorably, 143.1 to 123.2 mg/dL.

#### Bariatric Surgery

The current meta-analytic findings for overall T2DM remission (63.5 % [38.2–100.0]) and FPG reduction of 34.5 % (down to 95.3 mg/dL) after bariatric surgery are similar to those of a 2011 cross-sectional nRCT by Reed et al., in which bariatric surgery patients were observed prior to and at 1 week and 3 months following RYGB; FPG decreased to levels similar to those (≤125/mg/dL) of lean controls (BMI ≤25), and diabetes was considered resolved [71]. In the aforementioned SR of T2DM SG patients by Gill et al., a rate of 66.0 % remission was recorded [66], similar to our findings for the bariatric surgery group (63.5 %). In the 2004 SR/MA by Buchwald et al., diabetes was completely resolved in 76.8 % of bariatric surgery patients [5]; in the same authors’ 2009 SR/MA that focused on T2DM outcomes in 103 treatment arms (*n* = 3,188), at 2-year post bariatric surgery, complete diabetes resolution (defined therein as normal FPG and no anti-diabetic medications) was attained by 78.1 %; at ≥2 years, 74.6 % continued resolved [22]. An MA of low-BMI bariatric surgery patients by Li et al. reported a significant mean decrease in HbA_1C_ of 2.59 % in 80.0 % of patients after surgery, consonant with the decrease found in our meta-analysis (2.0 % [1.2, 2.8]) [72].

#### Conventional Treatment vs Bariatric Surgery

The overall T2DM remission rates for surgery patients vs conventional therapy patients presented in the current analysis were significantly different, 63.5 vs 15.6 % (*p* < 0.001), respectively. The relative efficacy of bariatric surgery and conventional therapy in promoting T2DM remission was further quantified in the meta-analytic calculation of PORs based on 16 studies, 94.0 % of which demonstrated a distinct statistical advantage in favor of bariatric surgery. When the analysis was run excluding trials reporting no remission events (which tend to produce inflated ORs), the summary point estimates were Peto POR of 6.9 (4.1, 11.6; inverse variance, 9.4 [5.0, 17.7], *p* < 0.001; *I* ^2^ = 75.4 %), still indicative of a large effect size favoring surgery. A second sensitivity analysis indicated that the odds of RYGB patients reaching T2DM remission were 8.0 times those of patients treated conventionally.

Remission event data were supported by parallel clinical evidence of T2DM remission in the form of HbA_1C_ outcome data. The pooled adjusted SMD quantifying the relative treatment effects of surgery vs conventional therapy on HbA_1C_ was 1.37 (1.01, 1.72), indicating that the average surgery patient experienced an HbA_1C_ outcome superior to that of 90.9 % of the conventional therapy group. RCT and OS study surgery patients experienced both statistically and clinically significant reduction in HbA_1C_, moving from baseline levels of 8.9 and 7.6 %, respectively, to 6.1 %, well below the ADA target of ≤7.0 %. Conventional therapy patients also experienced a statistically significantly reduction in HbA_1C_ relative to baseline; however, whether participating in an RCT or OS, this group continued to experience poor glycemic control (≥7.0 %) at follow-up. Glycated hemoglobin is a primary clinical marker and predictor of T2DM. Diabetic patients have an 11.0 % increased risk of mortality [73] from ischemic heart disease and, those with an HbA_1C_ >8 % are subject to a 150.0 % increased risk of death from heart disease [74]. Further, each 1.0 % increase in HbA_1C_ has been shown to be associated with a 20.0–30.0 % increase in cardiovascular events, and all-cause mortality independent of diabetes status [75].

The future will offer additional comparative evidence from high-quality, experimental studies of diabetes remission following bariatric surgery vs conventional treatment. A mid-2013 publication describes the start of a well-designed multicenter RCT of lower-BMI insulin-dependent patients who will undergo RYGB plus standard medical treatment, if needed, compared with controls who will receive only standard T2DM treatment; this RCT, the DiaSurg Trial, with a target size of 400 participants, will provide long-term comparative data (8 years) on T2DM outcomes following bariatric surgery vs conventional diabetes care [76].

#### Glycemic Control

Two studies in the current comparative meta-analysis provide reason to believe that the marked improvement in glycemic control following bariatric surgery is not only more pronounced than with conventional therapy, but more durable over the long term. Sjöström et al. reported a 96.0 % reduction in the risk of developing T2DM and a 36.0 % rate of T2DM remission maintenance in bariatric surgery patients at 10-year follow-up vs an *increase* in T2DM incidence in the usual care patient population [41]. Similarly, Iaconelli et al. reported 100.0 % prolongation of T2DM remission at 10-year follow-up vs 45.0 % in the medical therapy group [48]. In addition, a recently published follow-up report of the original, included, O’Brien et al. study [77] shows sustained weight loss and metabolic syndrome resolution after 10 years. All three of these long-term findings support those of Pories et al., who found >80.0 % improvement in T2DM maintained at 14 years post surgery [14], and of Li et al., whose MA of patients with <35 BMI showed several patients in whom diabetes resolution was maintained at 18 years following surgery [72].

#### Treating Lower-BMI Diabetic Patients

Multiple SR/MAs reveal a gradation in diabetes resolution with specific bariatric operations, wherein the greatest effect is associated with malabsorptive procedures, BPD and DS (95.0–98.9 %) [5, 22]. Yet, 90.0 % of T2DM patients are not morbidly obese [78–80]. The hypothesis of metabolic surgery—that certain procedures, some bariatric, performed without a primary weight-loss focus, but rather with the goal of achieving improved control or remission of diseases such as diabetes—has now been examined in several OSs, nRCTs, and RCTs [15, 58, 69, 81]. In our 2010 systematic review and integrative analysis of studies of metabolic surgery between 1979 and 2009 for treatment of T2DM in 343 patients with BMI <35, we reported 85.3 % T2DM resolution based on an FPG reduction of 93.3 mg/dL (105.2 mg/dL, −93.3) with patients off antidiabetic medications, and an HbA_1C_ reduction of 2.7 % into the normal range (<6.0 %) in addition to significant, not excessive, BMI loss of 5.1 (from 29.4 to 24.2) [15]. In a later review by Reis et al. that included our SR’s original 16 papers in addition to several additional more recently published studies, very similar results were found for patients with BMI <35 [82].

The current meta-analytic findings relevant to treating lower-BMI diabetic patients showed no significant heterogeneity between low- and high-BMI subgroups in summary estimates quantifying the relative effects of bariatric surgery vs conventional therapy on T2DM remission. The high-BMI (BMI ≥ 35) and lower-BMI (BMI < 35) subgroups had respective PORs of 15.2 (6.8, 34.1) and 17.1 (4.7, 62.9). This suggests that lower-BMI patients may also be able to experience the T2DM-reduction benefits of bariatric surgery long observed in higher-BMI patients.

Patients whose diabetes may be treated more effectively by bariatric surgery than conventional therapy, as found in the current meta-analysis that sought patients with a BMI ≥25, may benefit from the opportunity to elect T2DM treatment by surgery. The 2009 Asian Indian Consensus Statement, for example, recommended lowering the BMI cutoff for bariatric surgery with a comorbidity to 32.5 [83], making surgical treatment for diabetes available to more patients who, because of their ethnicity, may develop severe disease at lower weights than Caucasians [84]. Also, the 2011 International Diabetes Federation (IDF) Task recommendation for clinical practice as it relates to T2DM suggested that bariatric surgery should be considered an appropriate treatment under certain circumstances, including failure of conventional weight and T2DM therapy to control diabetes in those with a BMI of 30–35 [85]. Currently, however, the US National Institutes of Health (NIH) guidelines for bariatric surgery for patients with one or more comorbidity stipulate a BMI cutoff of ≥35, as do the Centers for Medicare and Medicaid Services (CMS), who maintain their 2006 policy of bariatric surgery approval only for patients with a BMI ≥35 [59, 86].

### Limitations

Only the available literature can be evaluated by a systematic review. The analytic power of this review was limited due to the diversity of T2DM diagnosis and remission standards reported. Most studies determined T2DM outcomes idiosyncratically; the majority of studies did not define T2DM remission uniformly, some employing ADA or NIH measures, and others, only biochemical marker of glycemic control. Although the number of available RCTs was noteworthy, representing almost one third of included studies, there is a shortage of well-controlled observational and level 1 (experimental) evidence comparing bariatric surgery and conventional therapy outcomes; a greater number of well-designed experimental studies would have increased this review’s predictive strength. A uniform standard for reporting T2DM remission is needed to improve the scientific evidence base and support clinical decision making. Another limitation of the analysis was that three studies, included because they met all comparative study requirements, incorporated conventional therapy arms that included no patient treatment, and simply followed patients who received no focused weight-loss intervention as a measure of “conventional treatment”.

Another possible limitation of this study was the use of standard deviation imputation in order to allow for inclusion of reported mean data that were not accompanied by variance data. In an effort to assess imputation bias, parallel analyses of between-group outcomes were carried out, incorporating into the meta-analyses only those studies with complete data, as originally abstracted. Without exception, imputation was shown to slightly constrain effect size. Also, the grouping of bariatric procedures may have introduced bias; however, sensitivity analysis demonstrated that the grouping technique, as applied in this research, also served to constrain effect size. Thus, SMDs reported herein likely represent relatively conservative summary estimates. Finally, our analyses afforded mainly short-term results: Comparative data for weight and T2DM remission in bariatric surgery vs conventional therapy at follow-up time points greater than 17.3 months are needed.

### Conclusions

The current research summarizes the best available findings for weight loss and T2DM remission in directly comparative research studies of surgery vs medical therapy in mildly obese, obese, and morbidly obese patients, a treatment effect corroborated by the analysis of HbA_1C_ reduction. The current review demonstrates that surgery has a dramatically greater weight-loss effect, by ≥3 orders of magnitude, and a more expeditious and higher rate of T2DM remission than conventional therapy.

Physicians must appraise the evidence for bariatric surgery vs conventional therapy on a patient-by-patient basis, weighing complications and costs associated with surgery against those associated with uncontrolled T2DM and/or comorbid obesity. However, only 1.0–3.0 % of individuals who reach class I obesity ever return to normal weight through conventional means [87], and a significant proportion of them go on to develop T2DM. Thus, the current meta-analytic finding, that the effectiveness of treatment with bariatric surgery far exceeds that of conventional therapy, carries the additional implication that surgery should be considered as a preventive measure.

In this systematic review and meta-analysis of 16 comparative studies of 6,131 patients with a baseline BMI ranging from 30.2 to 51.5 at mean 17-month follow-up, bariatric surgery was significantly more effective in achieving weight loss, HbA_1C_ reduction, and diabetes remission than conventional medical therapy. Based on overall summary estimates, the odds of T2DM remission in patients undergoing bariatric surgery were calculated to be from 9.8 to 15.8 times the odds of remission for those patients receiving conventional therapy, a finding that held true regardless of study design, severity of T2DM (as measured by baseline HbA_1C_), preoperative BMI, age, or weight loss.

## Electronic Supplementary Material

Below is the link to the electronic supplementary material.

ESM 1 DOCX 98 kb
