# Supplementary material for: Diabetes and Weight in Comparative Studies of Bariatric Surgery vs Conventional Medical Therapy: A Systematic Review and Meta-Analysis
Source: Obes Surg. 2013 Dec 28;24(3):437–55. doi: 10.1007/s11695-013-1160-3 (PMC3916703; doi:10.1007/s11695-013-1160-3)
Supplement: Supplementary file 1 — DOCX 98 kb [file 11695_2013_1160_MOESM1_ESM.docx]

**APPENDIX I**

*Statistical Analysis*

All data manipulation and analysis was conducted using SPSS^®^ software, version 20.0 (IBM SPSS, Chicago, IL) in conjunction with Comprehensive Meta Analysis 2.2 (Biostat, Englewood, NJ). Analysis of variance (ANOVA) was carried out on pooled baseline summary data characterizing RCT (surgery and conventional therapy) and OS (surgery and conventional therapy) groups. Patient characteristics and selected clinical variables of the surgical intervention group and the conventional therapy group were assessed for general baseline comparability by calculating pooled standardized mean differences (SMD) [37]. Within-group mean changes from baseline, as well as between-group mean differences along continuous outcome variables, were analyzed by calculating weighted mean differences (WMD) and associated 95% confidence intervals (95% CI). Treatment effect sizes were also estimated by pooling SMDs between groups and adjusting for corresponding variable baseline values.

Where included studies provided variable outcome means, but did not report associated standard deviations (SDs) or standard errors (SEs), SDs were imputed using methods adapted from Furukawa [38]. Parallel analyses of between-group outcomes using only studies with complete data were carried out as a check on potential imputation bias. Independent summary estimates were calculated for each study design subgroup (RCTs vs OSs) across quantitative and qualitative outcome variables. Concordance between RCTs’ combined magnitude of effect and OSs’ combined magnitude of effect was evaluated by heterogeneity testing and by noting degree of 95% CI overlap. Given the wide variation in study sample size, baseline BMI range, treatments, and outcome measures characterizing the included studies, random effects assumptions were applied throughout. To assess the validity of these assumptions, tests for heterogeneity were performed for each meta-analysis using the I^2^ statistic (calculated as: I^2^ = ([Q – *df*] / Q) x 100; where Q is the X^2^ statistic and *df* = degrees of freedom) [39].

Overall T2DM remission rates as well as mean percentage excess weight loss (%EWL) for the surgical intervention group and the conventional therapy group were calculated. In an effort to standardize reporting of overall weight loss, %EWL was calculated as: ([BMI reduction] / [baseline BMI – 25]) X 100. Statistical analysis of the relative effects of bariatric surgery vs conventional therapy on overall T2DM remission was carried out by aggregating remission event data and calculating the Peto pooled odds ratio (POR). The Peto method is assumption free and does not assume all studies included in the analysis are estimating the same treatment effect; in addition, corrections for zero cell counts are not necessary. In order to specifically evaluate whether differences in the summary odds estimates of RCTs vs OSs for T2DM remission were greater than what would be expected by chance alone, z-score calculation was performed using the following equation: z = (ln [POR_RCT_] – ln [POR_OS_]) / {Var (ln [POR_RCT_]) + Var (ln [POR_OS_])}^½^ [40] where ln [POR_RCT_] is the natural logarithm of the POR of RCTs assessing relative effectiveness of bariatric surgery vs conventional therapy on T2DM remission, and ln [POR_OS_] is the natural logarithm of the POR of OSs; and Var represents variance.

PORs and 95% CIs were also calculated using the more common inverse variance method for comparative purposes. Where cells contained zero (i.e., no events) within individual studies’ 2 X 2 odds ratio (OR) tables, the standard correction of adding 0.5 to each cell within a study’s contingency table was applied. The following sensitivity analyses were carried out: 1) an analysis excluding trials reporting no remission events in either arm; 2) an analysis excluding trials reporting outcome data from multiple arms; 3) an analysis using data solely from trials investigating the treatment effect of RYGB vs conventional therapy on T2DM remission. In addition to study design (RCT vs OS), several other pre-specified subgroup meta-analyses were conducted to explore potential sources of heterogeneity. Hypothesized categorical moderators included: age (<50 vs ≥50), baseline BMI (<35.0 vs ≥35.0) and HbA_1C_ levels (<8.0 vs ≥8.0), weight-loss differential (studies with <20.0% vs ≥20.0% BMI reduction difference), and sample size (<100 vs ≥100). Finally, publication bias was evaluated by the construction of a funnel plot of log Peto OR by SE, followed by the Begg-Mazumdar and Egger tests of asymmetry. All statistical tests were two-tailed and alpha was set at p<0.05. An I^2^ value of ≥75.0% was considered indicative of significant heterogeneity: A Q p-value of <0.10 was considered significant for heterogeneity.

**APPENDIX II**

*Sensitivity and Subgroup Analyses*

Three sensitivity analyses were performed. First, in an attempt to derive a conservative estimate of treatment effect, an analysis excluding trials reporting no remission events in either arm (k=11; n=1621) yielded a Peto POR of 6.9 (4.1, 11.6, <0.001; I^2^=78.5%) (Inverse variance: 9.4 [5.0, 17.7], p<0.001; I^2^=75.4%). The second sensitivity analysis excluded studies which combined data from multiple treatment arms (k=12; n=998), yielding a Peto POR of 10.5 (5.5, 20.3, <0.001; I^2^=81.1%) (Inverse variance: 19.3 ([7.4, 50.8], p<0.001; I^2^=79.3%). The third sensitivity analysis assessed data solely from trials (k=9; n=861) investigating the treatment effect of RYGB (the most frequently referenced bariatric procedure) vs conventional therapy on T2DM remission. The analysis yielded a Peto POR of 8.0 (3.7, 17.4, <0.001; I^2^=83.5%) (Inverse variance: 11.6 ([4.4, 30.7], p<0.001; I^2^=79.8%).

Finally, the moderating effects of age, baseline BMI and HBA_1C_ levels, weight-loss differential, and study size on the relative T2DM remission rates of BSG vs CTG patients were assessed via subgroup analysis. PORs and 95% CIs were calculated and are presented in **Table 5**. Although significant treatment effects favoring surgery were observed across subgroups (with inverse variance PORs ranging from 8.9–29.9), there was no significant heterogeneity (all Q p-values were nonsignificant) between subgroup estimates along any variable with the exception of sample size. Studies of low sample size (<100) had a significantly higher summary estimate of remission for the surgery group than did larger sample sizes (≥100) (27.2 [10.6, 68.8] vs 8.9 [3.7, 21.1], Q p-value<0.10).

*Publication Bias*

Visual inspectionof the funnel plot (**Fig. 6**) (SE by log Peto OR) revealed relative symmetry. This observation was supported by nonsignificant test of asymmetry (Begg Mazumdar test, Kendall’s tau b=0.08, p=0.65; Egger’s intercept: 1.2 [-1.4, 3.7], p=0.35). Thus, there was no immediate strong evidence of publication bias. However, given the comparatively small number of studies included (k=16) the statistical test suggestive of bias may not have been suffciently powered.

**APPENDIX III**

**Table 5.** Subgroup analyses of relative effects of surgery vs conventional therapy on diabetes remission

| **Subgroup** | **Number of studies** (N) | **Random effects**  **summary estimate**  (95% CI) | **P-value*** | **Q *P-value***† |
| --- | --- | --- | --- | --- |
| Age |  |  |  |  |
| High (≥50) | 5 (487) | 29.9 (7.5, 119.2) | <0.001 | NS (0.30) |
| Low (<50) | 11 (1406) | 12.8 (5.7, 28.8) | <0.001 |  |
| Baseline BMI |  |  |  |  |
| High (≥35) | 11 (1542) | 15.2 (6.8,34.1) | <0.001 | NS (0.88) |
| Low (<35) | 5 (351) | 17.1 (4.7, 62.9) | <0.001 |  |
| Baseline HbA_1C_ |  |  |  |  |
| High (≥8) | 6 (492) | 31.7 (6.6, 153.9) | <0.001 | NS (0.64) |
| Low (<8) | 6 (632) | 18.9 (4.4, 80.7) | <0.001 |  |
| Weight-loss differential**‡** |  |  |  |  |
| High (≥20.0%  BMI reduction  difference) | 8 (1282) | 22.1 (8.3, 58.7) | <0.001 | NS (0.40) |
| Low (<20.0%  BMI reduction  difference) | 8 (611) | 11.4 (4.3, 30.4) | <0.001 |  |
| Sample size |  |  |  |  |
| High (≥100) | 6 (1391) | 8.9 (3.7, 21.1) | <0.001 | <0.10 |
| Low (<100) | 10 (502) | 27.2 (10.7, 68.8) | <0.001 |  |

RCT, randomized controlled trial; OS, observational studies; BMI, body mass index; HbA_1C_, glycated hemoglobin; EWL, excess weight loss; Q, chi-squared statistic for heterogeneity.

*Test of overall effect, surgery vs conventional therapy.

†Test for heterogeneity of summary estimates between subgroups.

**‡**The “high” group represents studies with ≥20.0% BMI reduction between-group mean difference. The “low” group represents studies with <20.0% BMI reduction between-group mean difference.

**Fig. 6.** Funnel plot of log Peto odds ratio by standard error representing studies reporting remission data. The Effect size estimate for each study is plotted on the horizontal axis against standard error on the vertical axis.

**Figure 6**

**
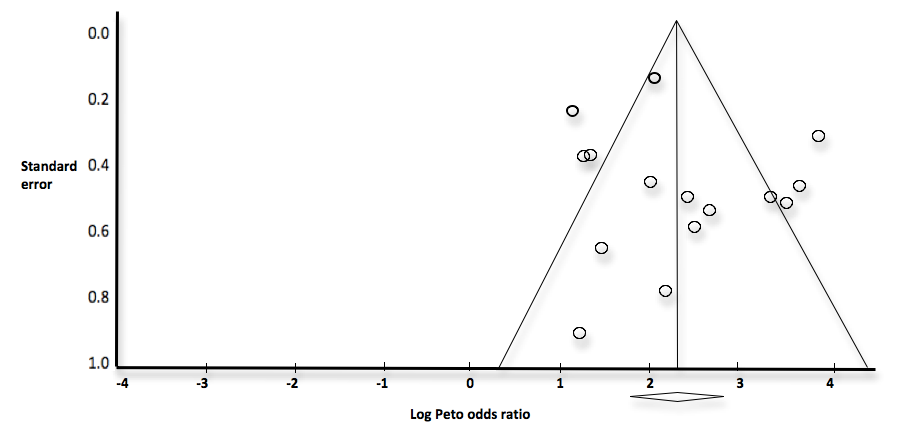
**
